# Validation Relaxation: A Quality Assurance Strategy for Electronic Data Collection

**DOI:** 10.2196/jmir.7813

**Published:** 2017-08-18

**Authors:** Avi Kenny, Nicholas Gordon, Thomas Griffiths, John D Kraemer, Mark J Siedner

**Affiliations:** ^1^ Last Mile Health Boston, MA United States; ^2^ Georgetown University Washington, DC United States; ^3^ Massachusetts General Hospital, Harvard Medical School Boston, MA United States

**Keywords:** data accuracy, data collection, surveys, survey methodology, research methodology, questionnaire design, mHealth, eHealth

## Abstract

**Background:**

The use of mobile devices for data collection in developing world settings is becoming increasingly common and may offer advantages in data collection quality and efficiency relative to paper-based methods. However, mobile data collection systems can hamper many standard quality assurance techniques due to the lack of a hardcopy backup of data. Consequently, mobile health data collection platforms have the potential to generate datasets that appear valid, but are susceptible to unidentified database design flaws, areas of miscomprehension by enumerators, and data recording errors.

**Objective:**

We describe the design and evaluation of a strategy for estimating data error rates and assessing enumerator performance during electronic data collection, which we term “validation relaxation.” Validation relaxation involves the intentional omission of data validation features for select questions to allow for data recording errors to be committed, detected, and monitored.

**Methods:**

We analyzed data collected during a cluster sample population survey in rural Liberia using an electronic data collection system (Open Data Kit). We first developed a classification scheme for types of detectable errors and validation alterations required to detect them. We then implemented the following validation relaxation techniques to enable data error conduct and detection: intentional redundancy, removal of “required” constraint, and illogical response combinations. This allowed for up to 11 identifiable errors to be made per survey. The error rate was defined as the total number of errors committed divided by the number of potential errors. We summarized crude error rates and estimated changes in error rates over time for both individuals and the entire program using logistic regression.

**Results:**

The aggregate error rate was 1.60% (125/7817). Error rates did not differ significantly between enumerators (*P*=.51), but decreased for the cohort with increasing days of application use, from 2.3% at survey start (95% CI 1.8%-2.8%) to 0.6% at day 45 (95% CI 0.3%-0.9%; OR=0.969; *P*<.001). The highest error rate (84/618, 13.6%) occurred for an intentional redundancy question for a birthdate field, which was repeated in separate sections of the survey. We found low error rates (0.0% to 3.1%) for all other possible errors.

**Conclusions:**

A strategy of removing validation rules on electronic data capture platforms can be used to create a set of detectable data errors, which can subsequently be used to assess group and individual enumerator error rates, their trends over time, and categories of data collection that require further training or additional quality control measures. This strategy may be particularly useful for identifying individual enumerators or systematic data errors that are responsive to enumerator training and is best applied to questions for which errors cannot be prevented through training or software design alone. Validation relaxation should be considered as a component of a holistic data quality assurance strategy.

## Introduction

A cornerstone of research conduct is the assurance of high-quality data collection. Data quality has been defined as “data that are fit for use by data consumer” [1]. Agmon and Ahituv [2] refer to data quality in terms of “reliability,” distinguishing between internal reliability (reliability whose assessment is based on commonly accepted criteria about the characteristics of the data items), relative reliability (reliability of the data in view of the user requirements), and absolute reliability (comparisons between the dataset and reality). Wand and Wang [3] take an ontological approach to identify 4 generic observable data quality issues—loss of information, insufficient (ambiguous) information, meaningless data, and incorrect data. If evidence is generated from underlying data that are of poor quality, incorrect conclusions may be drawn [4,5], leading to both direct and hidden costs [6,7].

The use of mobile phones and tablets for data collection may yield improvements over paper-based methods across a number of data quality dimensions and has been increasingly used in low-income settings [8-14]. Potential advantages of electronic methods over paper-based methods include lower error rates [10,13], reduced likelihood of data loss [8], higher data completeness [9,10,13], reduced time needed for data collection [9,10,13,15], automatic collection of timestamps and geolocation data, and in some cases decreased costs [9,13,16]. Additionally, electronic data collection has been shown to be feasible among users with little to no prior experience with data collection or cell phone use in a number of different settings, provided that they are given some basic training [8,9,12], and has been largely seen as acceptable by managers, users, and data collection subjects [9,12,13,16,17]. Thus, it represents an attractive option for researchers, nongovernmental organizations, governments, and others.

Claims of reduced error rates with mobile data platforms over paper alternatives can be logically attributed to several factors. Programmed skip logic (also called “branching”) allows for a question or data element to be displayed or not displayed depending on the user’s entry for 1 or more previous data elements, allowing for complex conditional pathways to be automated. This ensures that the proper sequence of questions or data elements are answered, ameliorating the problem of missing data. Real-time validation, notably the use of field constraints, is a restriction of the range or type of possible entries for a data element, limiting entries based on logical rules or previously entered data. This is widely viewed as a strong and appropriate tactic for reducing errors [18] in survey work, as it prevents the entry of logically invalid data. Furthermore, with electronic data collection, there is no manual data entry of paper forms needed, and thus the layer of errors associated with the manual data entry of paper data [19] is completely eliminated.

It has been recognized that data loss is still possible [12] and reductions in data quality have not been seen universally [15]. However, a challenge specific to electronic data collection that has not been explicitly addressed in the data quality literature is “masking” of data recording errors. Masking occurs when an end-user intentionally or unintentionally enters incorrect data that is forced or allowed by the data validation constraints. For example, an insufficiently trained user of a data collection application *without* hard-coded validation rules is likely to enter data that is illogical or internally inconsistent. However, if validation rules *are* applied, the data entered by such a user might still be susceptible to errors, but it will conform to the validation constraints, and thus such errors would not be detectable in the resulting dataset. When such errors could be mitigated by identification, supervision, and retraining, enabling errors to be rapidly identified and addressed is valuable.

In terms of Agmon and Ahituv’s dimensions of reliability, electronic data collection has great potential to increase internal reliability, as data constraints can be enforced; however, given the issue of masking, this will not always translate to increased absolute reliability. Similarly, in terms of Wand and Wang’s observable data quality issues, the problems of loss of information and meaningless data will be mitigated or eliminated, but this will only partially address the problem of incorrect data. As such, there is an important need to consider alternative methods of data quality oversight for mobile health data collection platforms.

In this paper, we articulate a strategy for assessing the data quality of electronic data collection initiatives by identifying incorrect data, thereby allowing for judgments on absolute reliability. This strategy, which we term *validation relaxation*, involves the intentional omission of validation features for a selection of data elements on which validation would typically be applied in order to allow for the possibility of detectable human errors, along with the creation of a mechanism for monitoring error rates and their trends in real time. Benefits of this approach include identification of instrument comprehension issues, detection of survey or database design errors, and targeted quality improvement efforts for individuals or teams with the highest error rates. We illustrate and evaluate an application of this strategy by describing its use within a cluster sample population survey in rural Liberia.

## Methods

### Development of the Validation Relaxation Strategy

The validation relaxation strategy was conceived to augment quality assurance of digital survey data collection operations at Last Mile Health, a nongovernmental health care organization operating in rural Liberia. The prior quality assurance approach contained 3 primary components: First, thorough training for survey enumerators, including observed survey practice with frequent instructor feedback and a field-based pilot test. Second, direct observation of a sample of surveys during the data collection period by a field supervisor, along with daily debriefings of field teams to review commonly committed errors. Third, the use of real-time validation and automated skip logic to prevent missing data and avoid illogical or impossible responses.

This approach was based on the Total Data Quality Management methodology, which emphasizes quality checks at multiple time points throughout the data life cycle [20]. The first quality component is a ubiquitous best practice in survey research [21]. The second is straightforward and has been employed in a variety of settings [22]. The third is seen as a major advantage of electronic data collection and has been leveraged extensively, often through the native capabilities of common data collection software packages [23-25] and sometimes through complex software customization that allows for the enforcement of idiosyncratic workflows [9]. However, as mentioned above, this third component can also mask underlying errors and lead to the production data that deceivingly appears clean.

To account for this issue, we created the “validation relaxation” strategy to detect intentional or accidental misuse of electronic data collection applications and avoid collecting poor-quality data. Specifically, we identified select scenarios in which human error can cause data to be collected that is logically valid but factually incorrect with electronic data collection. For example, if enumerators do not comprehend or administer an application correctly, they may intentionally falsify data to conform to data validation structures, an issue that has been previously considered in the context of survey-based research [26]. The validation relaxation strategy was intended to identify such instances by selectively removing form validation to allow for the possibility of unconstrained data entry, therefore making potential misunderstanding or misuse of the application quantitatively detectable, and subsequently monitoring error types and rates. Since only a sample of questions have validation rules removed, the overall *detectable* error rate for a given user may be thought of as a proxy measurement for the overall *undetectable* error rate, although the extent to which these rates correlate within a given set of users may vary between applications. Subsequently, focusing supervision and coaching efforts on the enumerators with the highest error rates may lead to decreases in overall error rates over time. Additionally, if the same survey instrument is used more than once (eg, in a repeated survey series), aggregate error rates can be used as an indicator of overall data quality differences between surveys.

To implement this strategy, we first created the data collection questionnaire and planned a set of validation rules including skip logic and field constraints to be applied, such that logically invalid responses and response patterns were prohibited by the application. We subsequently chose a purposive selection of 11 questions, out of a total of 122 survey questions, for which we removed (or “relaxed”) validation rules; this resulted in 11 different possible errors per survey. Questions were selected based on several factors; we were more likely to select questions for which we suspected or found data quality issues in the past (eg, dates), as well as questions that were relatively less important in the context of our ongoing research (to avoid compromising critical data during this evaluation). We also searched opportunistically for questions or sets of questions that allow for a logical rule to be easily validated (eg, the question “Have you ever given birth?” was already asked twice in the questionnaire to facilitate skip logic flow).

**Table 1:** Classification of detectable errors.

#	Class	Description	Example of error detected
1	Removal of “required” constraint	Removal of a “required question” constraint	User accidentally skips a question on postnatal care that he or she was supposed to complete
2	Illogical response combinations: multiple questions	Inclusion of 2 or more questions for which a certain combination of answers is logically impossible	The first question is “What is your gender?”; user answers “male.” The second question is “Have you ever given birth”; user answers “yes.”
3	Illogical response combinations: single question	Inclusion of an individual, multiple-response, multiple-choice question for which certain combinations of responses is logically impossible	The question is “Who checked on you during your last pregnancy?” User selects 2 options: “family members” and “I don’t know.”
4	Intentional redundancy	Repetition of the same question (possibly with slightly different wording or within a different question sequence) more than once in different sections of the questionnaire	At the start of the survey, user answers the question “How many times have you given birth?” with “6.” Later in the survey, the user answers a repeated instance of the same question (“How many times have you given birth?”) with “5.”
5	Manual skip logic	Forcing the user to select the next branch of questions to ask, based on responses to previous questions (instead of automating skip logic)	User answers the question “Have you ever been to a health clinic?” with a “No”. User is then prompted with 2 possible options and has to choose one: “Complete clinical questionnaire” or “Skip clinical questionnaire and proceed to child health questionnaire.” User selects “Complete clinical questionnaire.”
6	Removing minimum or maximum constraints	Removing constraints on the minimum or maximum value that can be entered for a question	User answers “657” to the question “How old are you, in years?”
7	Manual calculation	Prompt the user to enter a value that could be mathematically calculated from previous responses	Survey date is “June 3, 2016.” User answers the question “What is your birthday?” with “June 4, 1996.” The next question is “What is your age, in years?”; respondent answers “24.”
8	Allowing invalid data type	User is allowed to enter a value of an incorrect data type	The question is “How many times have you seen a doctor in the past month?” User answers “sometimes.”

We built and thoroughly tested the application, first in the office using a simulated dataset, and then through a field-based pilot test conducted in conditions that approximated the actual conditions in which the application was to be deployed. We created a reporting system to enable active monitoring of errors, disaggregated by the survey date and the enumerator’s ID number, which took the form of an automated report within a custom-built Web application written in the PHP (PHP: Hypertext Preprocessor) programming language.

After the implementation of the survey, we created a classification scheme of detectable errors to help facilitate the future selection of questions on which to relax validation. Detectable errors can be categorized based on the types of data elements under examination and the nature of the error that is permitted. This classification is detailed in Table 1.

### Data Collection

We assessed the validation relaxation strategy during the implementation of a 2-stage, cross-sectional, cluster sample survey in Rivercess County, Liberia. This was the second survey in a repeated cross-sectional study. Full description of the methods and results from the baseline survey has been described elsewhere [27]. The purpose of the survey was to assess a number of indicators of demographics, maternal health, neonatal health, and child health, as part of ongoing research and evaluation activities of Last Mile Health. The questionnaire was composed of questions adapted from the 2013 Liberia Demographic and Health Survey. Survey data were collected weekly from enumerators in the field by a supervisor and transferred to a secure, cloud-hosted MySQL database.

A total of 7 enumerators were hired to conduct the survey; each received a 5-day training covering the use of the data collection hardware and software, the purpose and meaning of each survey question, field translation in Bassa (the local dialect), and methods to reduce biases. An enumerator served as an alternate and only surveyed 10 women; data from this enumerator were excluded from this analysis.

The platform used was a modified version of Open Data Kit (ODK), an open-source set of tools designed to allow implementers to create information systems in the developing world [23]. Modifications to ODK allowed for data to be transferred wirelessly from one Bluetooth device to another, which was advantageous for prevention of data loss, given that Liberia’s poor cellular network coverage meant that users would be out of coverage for many consecutive days. Our modified ODK application was installed on 10 BLU Advance 4.0 Android phones, which were distributed to enumerators and field supervisors. Data collected on the Android devices were stored in XML format, transferred periodically from enumerator phones to supervisor phones via Bluetooth, and ultimately transferred via Bluetooth to a central laptop, where records were uploaded to a custom-built Web application. This application parses the data into JSON (JavaScript Object Notation) format, checks for file integrity, adds several metadata attributes, and sends the resulting dataset into a MySQL database cloud-hosted on a virtual private server.

Enumerators were not informed of the validation relaxation strategy. During the implementation of the survey, we ran the automated error report on a weekly basis, which was used to identify enumerators who were underperforming, as evidenced by high error rates relative to the other enumerators. Each week, the lead field supervisor of the survey examined error rates and focused monitoring and coaching efforts on underperforming enumerators.

### Data Analysis

Error rates were summarized using basic descriptive statistics. We then used logistic regression to estimate the association between time (survey day) and the odds of committing an error. No covariates were included in the model. Variance estimation was corrected for the effects of clustering using the clustered sandwich estimator. Next, we collapsed the dataset such that 1 observation represented a single survey day and estimated the daily standard deviation of error rates, and used linear regression to estimate the association between time (survey day) and the standard deviation of error rates between enumerators. Statistical analyses were conducted using Stata Version 14.1 (Statacorp).

### Ethics

Ethics approval for the survey was obtained from the institutional review boards of Partners Healthcare, Georgetown University, and the Liberian Institute for Biomedical Research. All respondents gave verbal informed consent.

## Results

The survey was conducted between April 12, 2016 and June 7, 2016, and included a sample of 972 women across 1150 households within 86 different communities. Table 2 details the specific errors that were possible within our survey, along with error rates for each. For the calculation of rates, the denominator is equal to the number of times that the requisite question(s) were reached within the application by the enumerator. In other words, the rate is equal to the number of errors divided by the number of opportunities for the error to be made.

The overall error rate was 1.60% (125/7817). This is comparable to error rates in similar settings [28,29]. The most commonly made error was an “intentional redundancy” question in which the respondent was asked twice for the date of birth of her most recently birthed child, with an error rate of 13.6% (84/618). Data for this question were entered through an ODK “date widget” [30], where the enumerator scrolls through month, days, and years to select the correct date. An examination of incorrect dates suggests that the high rate for this particular error may have partially been due to the enumerator accidentally scrolling 1 or 2 ticks past the correct day, month, or year; 30% (25/84) of these errors were 1 tick off and an additional 17% (14/84) were 2 ticks off. However, it is also possible that the respondent’s recall is inexact. Four other possible errors had rates between 0.2% and 3.1%, with all other possible errors having rates equal to zero. There was a strong association between the class of the error and the rate at which the error was committed. Specifically, the 4 intentional redundancy errors had the 4 highest error rates.

**Table 2:** Specific detectable errors implemented in cluster sample survey.

#	Class	Error definition	Number of errors	Error rate, %
1	Intentional redundancy	Gave different answers for the question (“Was your most recent birth in a health facility?”) in different sections of the questionnaire	19/618	3.1
2	Intentional redundancy	Gave different answers for the question (“Have you ever given birth?”) in different sections of the questionnaire	10/961	1.0
3	Intentional redundancy	Gave different answers for the question (“What was the date of birth of your most recently birthed child?”) in different sections of the questionnaire	84/618	13.6
4	Intentional redundancy	Gave different answers for the question (“Is your most recently birthed child still alive?”) in different sections of the questionnaire	10/618	1.6
5	Illogical response combinations: single question	Question is “Where you go to get medical advice or treatment?”; answer options included (“refused to respond” OR “unknown”) AND (“clinic” OR “drugstore” OR “community health worker” OR “traditional healer” OR “other”)	2/895	0.2
6	Illogical response combinations: single question	Question is “What are the signs of someone who can have ebola?”; answer options included (“refused to respond” OR “unknown”) AND (“fever” OR “muscle pains” OR “vomiting” OR “sore throat” OR “diarrhea” OR “bleeding” OR “other”)	0/895	0.0
7	Removal of “required” constraint	A required question (“Can people get Ebola from touching an Ebola patient?”) was skipped	0/895	0.0
8	Removal of “required” constraint	A required question (“Can people get Ebola from the air?”) was skipped	0/895	0.0
9	Removal of “required” constraint	A required question (“Can people get Ebola by touching or washing a dead body?”) was skipped	0/895	0.0
10	Illogical response combinations: multiple questions	Answers for a multiple-response question (“From whom did the child get treatment [for fever or cough]?”) were given; an answer was given to the following question (“From whom did the child get treatment FIRST?”) that was not selected in the previous list of responses	0/325	0.0
11	Illogical response combinations: multiple questions	Answers for a multiple-response question (“From whom did the child get treatment [for diarrhea]?”) were given; an answer was given to the following question (“From whom did the child get treatment FIRST?”) that was not selected in the previous list of responses	0/202	0.0
		Total	125/7817	1.60

Roughly twice per week during the survey implementation, the lead field supervisor reviewed an error report that summarized errors committed so far, disaggregated by the survey date and the enumerator ID number. During the survey data collection period, this report was accessed on 18 different days by the supervisor (with a roughly uniform distribution), based on database usage tracking statistics. The supervisor would then communicate with the 2 other field supervisors, and give the names of the enumerators with high error rates, along with information on which errors were being commonly made. Total enumerator-specific error rates are summarized in Table 3.

Differences between enumerators were not statistically significant for any of the time periods evaluated. An analysis of variance of the data in each of the 4 columns in Table 3 gives the *P* values given in the bottom row of the table.

**Table 3:** Enumerator-specific error rates.

Enumerator ID#	Error rate, %			Overall error rate, %
	(day 0-14)	(day 15-29)	(day 30-45)	(day 0-45)
2	3.1 (14/458)	0.4 (2/465)	1.2 (5/415)	1.57 (21/1338)
3	2.9 (13/452)	0.8 (3/382)	1.8 (6/334)	1.88 (22/1168)
4	2.8 (17/605)	1.8 (9/506)	1.3 (5/393)	2.06 (31/1504)
5	1.8 (7/386)	1.6 (6/364)	1.0 (3/286)	1.54 (16/1036)
6	2.5 (14/552)	0.9 (5/528)	0.2 (1/436)	1.32 (20/1516)
7	1.4 (7/512)	1.6 (6/380)	0.6 (2/363)	1.20 (15/1255)
	*P*=.45	*P*=.45	*P*=.42	*P*=.51

Data quality improved over time. A logistic regression of time on the error variable (a binary variable representing whether an error was committed) was significant (*P*<.001), with an odds ratio of 0.969 (95% CI 0.955-0.983), representing the change in odds given a 1-day change in time. Thus, the predicted total change in average error rate from the start of the survey (day=0) to the end (day=45) is −1.7%, representing a fourfold decrease in error rate, from 2.3% (95% CI 1.8%-2.8%) to 0.6% (95% CI 0.3%-0.9%) over the observation period. Data are summarized in Table 4.

**Figure 1:**
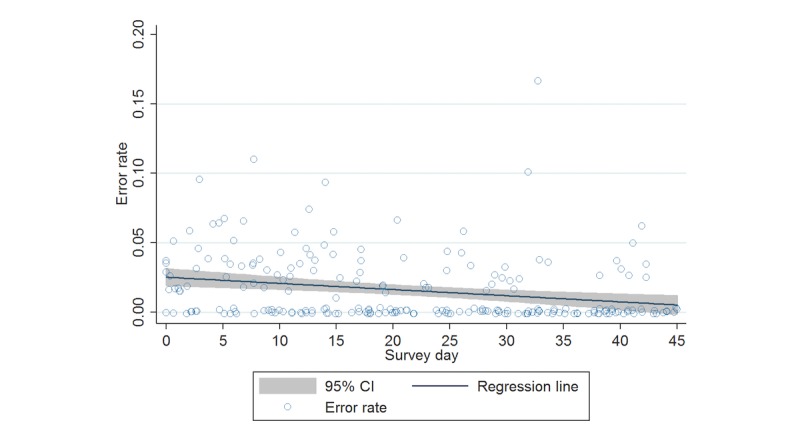
Daily enumerator-specific error rates over time, with fitted regression line (jittered for clarity).

Data for sensitivity analysis #4 (similar to primary analysis #1, except leveraging aggregated data) are visualized in Figure 1.

**Table 4:** Change in error rates over time (primary and sensitivity analyses).

Analysis	Type	Number of observations	Odds ratio (OR) or coefficient (beta) (95% CI)	*P* value	Predicted error rate at day=0 (95% CI)	Predicted error rate at day=45 (95% CI)
Primary (#1); all errors included	Logistic regression	9527	OR=0.969 (0.955 to 0.983)	<.001	0.0230 (0.0179 to 0.0281)	0.0056 (0.0027 to 0.0085)
Sensitivity (#2); excludes most common error	Logistic regression	8566	OR = 0.985 (0.964 to 1.007)	.18	0.0064 (0.0041- to 0.0086)	0.0032 (0.0010 to 0.0055)
Sensitivity (#3); includes only 3 most common errors	Logistic regression	2883	OR = 0.965 (0.949 to 0.982)	<.001	0.0710 (0.0512 to 0.0908)	0.0153 (0.0059 to 0.0248)
Sensitivity (#4); includes only 5 most common errors; aggregated data	Logistic regression	4739	OR = 0.968 (0.954 to 0.982)	<.001	0.0461 (0.0356 to 0.0567)	0.0112 (0.0055 to 0.0168)
Sensitivity (#5); all errors included; aggregated data	Linear regression	218	beta = −.000444 (−.000607 to −.000280)	<.001	0.0252 (0.0189 to 0.0315)	0.0052 (−0.0007 to 0.0111)
Sensitivity (#6); excludes most common error; aggregated data	Linear regression	218	beta = −.000051 (−.000171 to −.000070)	.33	0.0069 (0.0048 to 0.0091)	0.0047 (−0.0008 to 0.0101)
Sensitivity (#7); includes only 3 most common errors; aggregated data	Linear regression	218	beta = −.002235 (−.003353 to −.001118)	.004	0.1094 (0.0723 to 0.1465)	0.0088 (−0.0186 to 0.0361)
Sensitivity (#8); includes only 5 most common errors	Linear regression	218	beta = −.000903 (−.001256 to −.000549)	.001	0.0530 (0.0399 to 0.0662)	0.0124 (−0.0032 to 0.0281)

## Discussion

### Principal Findings

We describe the development and evaluation of *validation relaxation*, a novel strategy that involves the intentional omission of electronic data collection validation features for a selection of data elements to allow for the possibility of detectable human errors, which enables data error rate monitoring and identification of database design and survey comprehension issues. We evaluated this strategy in the field during a population survey in rural Liberia, and found that date question formats were the most problematic, and that error rates were largely consistent between enumerators, and that error rates decreased significantly over time.

This strategy enabled us to learn what types of errors were most commonly occurring and implement training measures to ensure optimal use and comprehension of the data collection platform and survey instrument, respectively. The overall error rate was low at 1.60%, and although error rates did not differ significantly between enumerators, they varied considerably between error types. The highest error rates were found for the “intentional redundancy” errors. There are several possible reasons for this trend. First, 3 of the 4 intentional redundancy questions were grouped in one of the most complicated survey sections in terms of the underlying skip logic. Second, there may have been higher error on the part of the respondents, as they were asked about events that often occurred many years ago. Third, the highest error rate was detected for a date question, and as discussed, the date selector widget was prone to accidental error if the user scrolled too far, resulting in a higher probability that the incorrect value was entered.

### Applications

We assessed the validation relaxation strategy during a survey in a low-income setting, but the strategy may also have value across other data collection scenarios including research studies, electronic medical record systems, and mHealth/eHealth initiatives in both developing and high-income settings. It should be considered in addition to other emerging electronic data quality improvement techniques, such as automatic filling of forms [31,32] and dynamic reordering and repeating questions [33], as an additional method to optimize data quality for electronic data collection. Similarly, although we employed validation relaxation to compare error rates between multiple users, it can also be a useful means of assessing trends in data quality. It can also be potentially useful in comparing enumerator or field teams who are individually and simultaneously implementing a data collection instrument. Automated or semiautomated feedback loops can be employed with this strategy to enable real-time detection of errors, which can be used to intervene on faulty survey instruments or to improve enumerator data collection quality [8,34].

Validation relaxation might also allow data managers to detect fraud in data collection applications. Existing approaches to fraud detection focus on conducting repeat interviews for a sample of respondents [35], identifying “at-risk” enumerators [36], examining digit preference (Benford’s Law) [37,38], analyzing the statistical qualities of specific variables within the dataset [37,39], leveraging machine learning algorithms to detect anomalies in response distribution [40], and searching for patterns in survey timestamps [8]. The inclusion of intentionally redundant questions, preferably spaced apart within a questionnaire, could lead to patterns of inconsistent response for a single user, which would signal a possible case of falsification.

Finally, although its initial intent was to identify end-user data entry errors, validation relaxation might also help detect errors in application/database design. Often, designers will make assumptions about the potential set of logical response options (eg, an enumerator trying to enter the value “14” on a question that asks for the age of a pregnant woman, where the input range is restricted to 15-49 years). By relaxing validation rules, designers can remove such assumptions regarding valid data ranges, empirically test whether the actual range of collected values falls within the expected range, and subsequently investigate records where values fall outside the expected range.

### Limitations

This work was limited to quantitative assessment of the strategy. Future work should include qualitative input from database designers and end-users to further explore the nature of committed errors and enumerator perceptions of the strategy. More data are also needed to better specify the large-scale feasibility and cost of this strategy if applied to large health programs. Moreover, our hypothesis that the *detectable* error rate is a good proxy measurement for the *undetectable* error rate is an assumption that warrants further investigation. Our list of error types and validation domains were self-selected based on our own experience and hypothesis and future iterations of this technique can and should expand upon these to target a more thorough and case-specific error types and validation schemes. Other possible alterations to the strategy to consider for future use include prespecification of a maximum acceptable error rate, use of control charts [41], and use of a formal statistical test to determine whether or not error rates between enumerators or surveys significantly differ.

### Conclusions

The validation relaxation strategy can help detect comprehension and platform usability issues for electronic data collection applications, detect end-user and program error rates, and elucidate trends in error rates over time or between user groups. The strategy should be implemented as one component of a holistic data quality approach in the increasingly widespread use of electronic data collection platforms.

## Abbreviations

JSON: JavaScript Object Notation
ODK: Open Data Kit
PHP: PHP: hypertext preprocessor
